# Natural Bagaza virus infection in game birds in southern Spain

**DOI:** 10.1186/1297-9716-43-65

**Published:** 2012-09-11

**Authors:** Virginia Gamino, Ana-Valeria Gutiérrez-Guzmán, Isabel G Fernández-de-Mera, José-Antonio Ortíz, Mauricio Durán-Martín, José de la Fuente, Christian Gortázar, Ursula Höfle

**Affiliations:** 1 Instituto de Investigación en Recursos Cinegéticos IREC, (CSIC-UCLM-JCCM), Ronda de Toledo s/n, 13071, Ciudad Real, Spain; 2 Centro Vigilancia Sanitaria Veterinaria (VISAVET), Departamento de Sanidad Animal, Facultad de Veterinaria, Universidad Complutense de Madrid, Madrid, Spain; 3 Medianilla S.l, Benalup, Cádiz, Spain

## Abstract

In late summer 2010 a mosquito born flavivirus not previously reported in Europe called Bagaza virus (BAGV) caused high mortality in red-legged partridges (*Alectoris rufa*) and ring-necked pheasants (*Phasianus colchicus*). We studied clinical findings, lesions and viral antigen distribution in naturally BAGV infected game birds in order to understand the apparently higher impact on red-legged partridges. The disease induced neurologic signs in the two galliform species and, to a lesser extent, in common wood pigeons (*Columba palumbus*). In red-legged partridges infection by BAGV caused severe haemosiderosis in the liver and spleen that was absent in pheasants and less evident in common wood pigeons. Also, BAGV antigen was present in vascular endothelium in multiple organs in red-legged partridges, and in the spleen in common wood pigeons, while in ring-necked pheasants it was only detected in neurons and glial cells in the brain. These findings indicate tropism of BAGV for endothelial cells and a severe haemolytic process in red-legged partridges in addition to the central nervous lesions that were found in all three species.

## Introduction

In late summer 2010 an extremely high mortality was observed among game birds in southern Spain, especially in red-legged partridges (*Alectoris rufa*), but also in ring-necked pheasants (*Phasianus colchicus*), that was determined to be due to a flavivirus not previously reported in Europe, BAGV [1]. BAGV is a relatively unknown member of the Ntaya group of the genus *Flavivirus* that was first isolated in the Central African Republic, in 1966, from a pool of *Culex* spp. mosquitoes [2].

Although a recent study has examined the epidemiology of the outbreak more closely [3], information on the pathogenesis of BAGV is very limited. Thus in the present study we review the main clinical findings, lesions and viral antigen distribution in wild birds naturally infected with BAGV. Moreover we compare these features among BAGV-infected red-legged partridges, ring-necked pheasants and common wood pigeons (*Columba palumbus*), in order to understand the pathogenesis of the disease and the reason for the extreme impact of the disease in red-legged partridges in comparison to other affected species.

## Material and methods

### Study area

The study area is located on the Mediterranean coast, in south-western Spain (between 36° 20’ N and 5° 48’ O). The climate is Mediterranean, with wet winters and dry summers. The habitat is a mosaic of intensively managed crops, primarily rice and vegetables, and open woodland (dehesa) with cork oaks (*Quercus suber*), and stone pines (*Pinus pinea*).

### Postmortem examination and sampling

Recently dead or moribund red-legged partridges (*n* = 6), ring-necked pheasants (*n* = 5) and common wood pigeons (*Columba palumbus*, *n* = 2) were collected in the area during three consecutive days. Oral and cloacal swabs were taken and stored in viral transport medium (Hanks’ balanced salt solution containing 10% glycerol, 200 U/mL penicillin, 200 μg/mL streptomycin, 100 U/mL polymixin B sulphate, 250 μg/mL gentamicin and 50 U/mL nystatin [4]) and frozen immediately in liquid nitrogen.

Detailed necropsies were carried out, and a complete set of tissues was taken (brain, oral mucosa, pectoral muscle, trachea, lung, heart, liver, spleen, pancreas, duodenum, caecal tonsils, kidney, bursa of Fabricius, thymus and skin with feather follicles) and fixed in 10% neutral buffered formalin for histopathologic examination. Additionally, samples of heart, kidney, brain and spleen were also collected into sterile containers and frozen immediately in liquid nitrogen.

### Real time RT-PCR and sequence analysis

Swabs and frozen tissue samples were processed upon arrival at the laboratory.

Nucleic acid (RNA) was extracted using High Pure RNA Tissue Kit (Roche Diagnostics, Barcelona, Spain), and analysed by real time RT- PCR for the presence of West Nile Virus (WNV) and flavivirus genome in general. For WNV detection, a TaqMan MGB multiplex real time RT-PCR using a commercial kit (QuantiTEC Probe® RT-PCR, Qiagen, Madrid, Spain) was applied as described before [5]. Flavivirus detection was carried out as described previously by generic SYBR Green (Qiagen, Madrid, Spain) real time RT-PCR [6].

A generic nested RT-PCR previously described [7] was used to confirm flavivirus detection. Reactions were performed using the Access RT-PCR System (Promega, Madison, WI, USA) in an automated DNA thermal cycler model TC-512 (Techne, Cambridge, UK). Briefly, 5 μL (around 10 νg) of nucleic acid preparation was added to 45 μL of a RT-PCR mix containing 2 mM MgSO_4,_ 0.2 mM of dNTP, 1× AMV/Tfl 5× reaction buffer, 5 U *Tfl* DNA polymerase, 5 U AMV reverse transcriptase and 40 pmols of each primer (Flavi1+ and Flavi1-) and amplified using an initial incubation at 38°C for 45 min followed by 94°C for 2 min, and 40 cycles of 94°C for 30 s, 47°C for 1 min, and 68°C for 1 min and 15 s. The nested PCR reaction was carried out in a final volume of 50 μL, with similar concentrations as in the first reaction, using primers Flavi2+ and Flavi2-, and 1 μL of the product of the first amplification. The mix was subjected to 94°C for 2 min, followed by 40 PCR cycles with similar conditions to those used in the primary generic RT-PCR.

Subsequently, 8 μL of each PCR product was subjected to electrophoresis on a 2% agarose gel to check the size of amplified fragments by comparison to a DNA molecular weight marker (1 kb Plus DNA Ladder, Promega, Madison, WI, USA).

The DNA bands from the nested and the first generic amplification were resin-purified (Wizard, Promega Madison, WI, USA) and cloned into pGEM-T (Promega, Madison, WI, USA). At least four independent clones were sequenced from both ends for each positive sample (Secugen SL, Madrid, Spain). Sequence similarity search was performed using BLAST [8].

### Histopathology and immunohistochemistry

Formalin-fixed tissues were trimmed, embedded in paraffin, sectioned at 4 μm and processed to obtain haematoxylin-eosin stained sections. These were examined independently by two different investigators (VG and UH).

Furthermore, the avidin-biotin-peroxidase complex (ABC) method was used on paraffin-embedded tissue sections for immunohistochemical demonstration of flavivirus antigen. For this purpose, a rabbit polyclonal antibody against WNV that has been shown to cross-react with other flaviviruses [9], was used (BioReliance, Product 81–015, Rockville, Maryland, USA). After deparaffinization and hydration of the sections, they were incubated with 3% H_2_O_2_ diluted in methanol for 30 min at room temperature (20–25°C) in order to block endogenous peroxidase activity. After rinsing with water, the sections were treated with proteinase K (Diagnostic BioSystems, Pleasanton, California) for 15 min at room temperature. They were then rinsed with water, followed by three 5 min rinses in 0.1% Tris-buffered saline/Tween20 (TBS 0.05 M, pH 7.5), and incubated 1 h at room temperature with 2% albumin from bovine serum (Sigma-Aldrich Chemie, Steinheim, Germany) diluted in 0.1% TBS/Tween20. The primary antibody was applied overnight at 4°C at a dilution of 1:1000 in 2% albumin-0.1% TBS/Tween20. After three 5 min rinses in 0.1% TBS/Tween20, sections were incubated with a biotinylated goat antirabbit IgG (Vector Laboratories, Burlingame, California, USA) diluted 1:200 in 0.1% TBS/Tween20 for 1 h at room temperature. After three 5 min rinses in 0.1% TBS/Tween20 an avidin-biotinylated enzyme complex (ABC system, Vector laboratories) was applied for 30 min at room temperature according to manufacturer’s recommendations. For development, sections were incubated for 1 min with 3,3Â´-diaminobenzidine tetrahydrochloride according to manufacturerÂ´s recommendations (Vector Laboratories). Sections were counterstained with MayerÂ´s haematoxylin. Negative controls included substitution of the primary antibody by 2% albumin from bovine serum diluted in 0.1% TBS/Tween20 and negative rabbit antibody (BioReliance, Product 81–015, Rockville, Maryland, USA). Tissue sections of experimentally infected red-legged partridges in which presence of WNV had been confirmed by real time RT-PCR served as positive controls.

## Results

### Morbidity and mortality

The outbreak affected mostly gallinaceous game birds and caused nervous signs and high mortality. Apparently the primary species affected by the outbreak were red-legged partridges and ring-necked pheasants. Carcasses and individuals displaying clinical signs of apparent blindness, ataxia and lack of coordination belonged primarily to these two species, although a low number of affected columbiformes (Common wood pigeon) had also been observed. Mortality or nervous signs in other avian species such as corvids or birds of prey were not detected.

### Real time RT-PCR and sequence analysis

All samples tested negative for genomes of WNV lineage 1 and 2 by real time RT-PCR. Flavivirus genome was detected by real time RT-PCR in all tested individuals in at least one of the samples analyzed (swabs and tissues, Table 1). A positive signal was obtained most frequently in the brain (67%) and oral swabs (67%) of partridges, and in the kidney (80%), and brain (100%) of pheasants. In one of the wood pigeons sampled, all tested tissues were positive for flavivirus, while only the kidney of the other one was positive and oral and cloacal swabs were negative in both individuals (Table 1).

**Table 1:** Detection of Flavivirus genome in BAGV infected game birds

**Sample**	**Partridge**						**Pheasant**					**Wood Pigeon**	
	**1**	**2**	**3**	**4**	**5**	**6**	**1**	**2**	**3**	**4**	**5**	**1**	**2**
Oral swab	19	−	30	23*	−	19*	−	−	29	31	−	−	−
Clocacal swab	−	−	−	−	−	29	−	−	−	31	−	−	−
Heart	−	−	32	23	−	23	−	−	−	−	27	31	−
Kidney	29	30	−	−	−	−	28	−	29	27	29	29	30
Brain	−	22*	22*	16*†	30	−	26*	23*	24*	30	30	30	−
Spleen	30	−	−	−	−	30	−	−	−	−	−	27	−

*Sequenced 104 bp amplicon from conventional PCR.

† Sequenced 1048 bp amplicon from conventional PCR.

− Samples that did not show amplification.

Detection of flavivirus genome by rtRT-PCR (Ct values) and by sequencing from conventional PCR in different tissues and swabs.

Only one partridge brain sample yielded a DNA product sufficient for cloning and sequencing in the primary conventional generic, nested flavivirus RT-PCR. In the sequence similarity search performed by using BLAST, the analyzed sequence of 1048 bp showed 92% sequence identity to previously reported Bagaza virus strain DakAr B209 (GenBank: AY632545.2). In the case of the nested PCR, the 104 bp amplicon was sequenced from 8 different samples; 3 brain samples and 2 oral swabs from partridges, and 3 brain samples from pheasants, showing 97-98% sequence identity to the previously described BAGV (Table 1).

### Gross and microscopic lesions

The distribution, type and severity of lesions varied considerably between species. Two partridges and two pheasants were emaciated. The most striking macroscopic lesions in partridges included pallor of the pectoral muscle (4/6), pancreas (4/6) and myocardium (3/6) and injection of encephalic (3/6) and epicardial (4/6) vessels. Besides thickening of the pericardium (2/5) or pallor of the myocardium (2/5), no significant gross lesions were observed in pheasants. Equally, no significant macroscopic lesions other than encephalic vessel injection (2/2) were found in wood pigeons.

**Figure 1:**
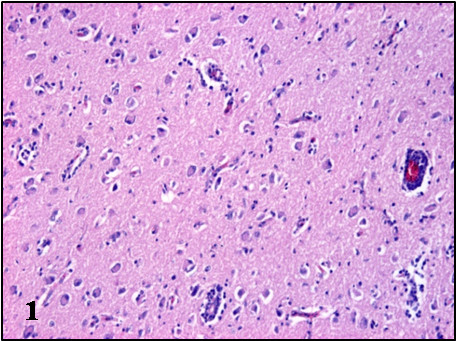
**Microscopic lesions in the brain of BAGV infected game birds.** Cerebrum, ring-necked pheasant. Neuronal necrosis and lymphoplasmacytic perivascular infiltrates. HE, ×100.

**Figure 2:**
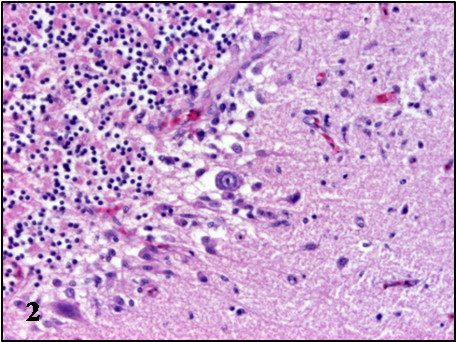
**Microscopic lesions in the brain of BAGV infected game birds.** Cerebellum, ring-necked pheasant. Purkinje cell necrosis and glial nodule. HE, ×200.

**Figure 3:**
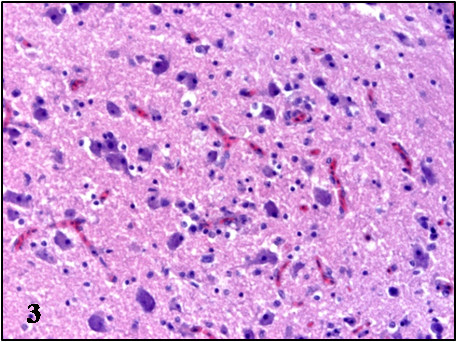
**Microscopic lesions in the brain of BAGV infected game birds.** Cerebrum, red-legged partridge. Neuronal necrosis and gliosis. HE, ×200.

**Figure 4:**
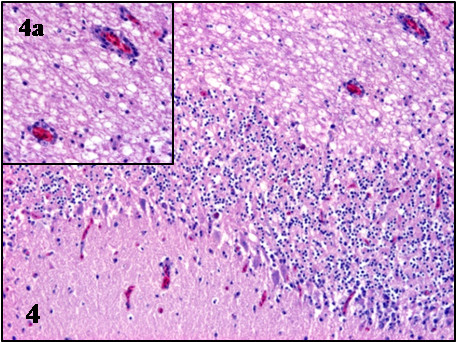
**Microscopic lesions in the brain of BAGV infected game birds.** Cerebellum, red-legged partridge. Purkinje cell necrosis and phagocitosis and endothelial cell swelling (**4a**). HE, ×100.

**Figure 5:**
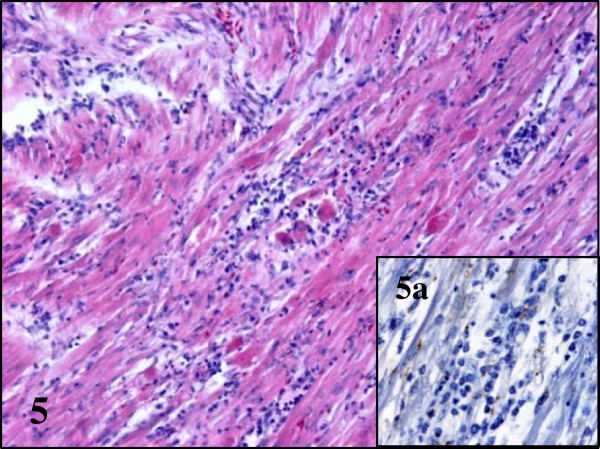
**Lesions and BAGV antigen distribution in the heart of BAGV infected red-legged partridges.** Severe myocarditis with presence of BAGV antigen labeling in myofibres and capillary endothelial cells (**5a**). HE, ×100; immunohistochemistry for detection of flavivirus antigen, ×400.

**Figure 6:**
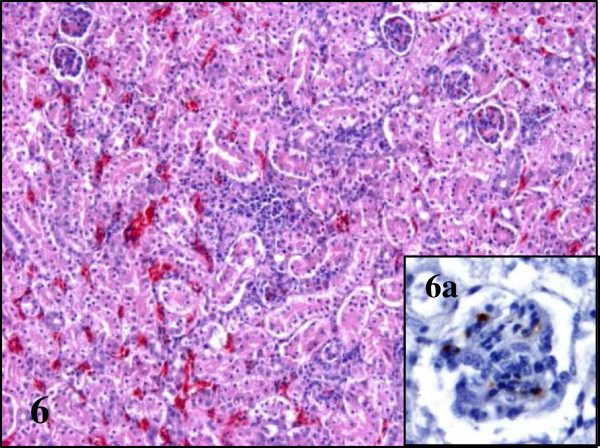
**Lesions and BAGV antigen distribution in the kidney of BAGV infected red- legged partridges.** Focal necrosis and BAGV antigen labelling in capillary endothelial cells of the glomerular mesangium (**6a**). HE, ×100, immunohistochemistry for detection of flavivirus antigen, ×400.

In all cases, the most prominent microscopic lesions were congestion, necrosis and mononuclear inflammatory infiltrates consisting of lymphoid cells, plasma cells and histiocytes, although to a different degree in individuals of the three species (Table 2). The most affected systems were the central nervous system and the spleen. Lesions in the brain were characterized by congestion, gliosis, neuronal necrosis and phagocytosis, perivascular cuffing, capillary endothelial cell swelling and Purkinje cell necrosis and disappearance. The perivascular infiltrates and glial nodules were constituted by lymphocytes, plasma cells and histiocytes and were mainly present in the gray matter of the cerebrum, the brain stem and molecular layer of the cerebellum (Table 2, Figures 1, 2, 3, 4). In the spleen, lymphoid depletion, necrotic foci of lymphoid cells, thickened capsule, and multifocal granulocytic infiltrates were evident. Other organs affected included the kidney, heart and liver, where necrosis and lymphoplasmacytic and histiocytic infiltrates were the most important lesions (Table 2, Figures 5 and 6).

**Table 2:** Microscopic lesions in BAGV infected game birds

**Lesion**	**Partridge**						**Pheasant**					**Wood Pigeon**	
	**1**	**2**	**3**	**4**	**5**	**6**	**1**	**2**	**3**	**4**	**5**	**1**	**2**
**Cerebrum**													
Neuronal necrosis	−	−	−	−	−	−	+	−	+	+	+	NT	+
Neuronophagia	+	−	+	−	+	−	+	−	−	−	+	NT	+
Satellitosis	+	+	+	+	−	−	−	−	−	+	+	NT	−
Gliosis	+	+	+	+	+	+	+	+	+	+	+	NT	+
Perivascular cuffing	−	−	−	+	−	+	+	+	+	−	−	NT	−
Endothelial hypertrophy	+	+	+	+	+	+	+	+	+	+	+	NT	+
Glial necrosis	−	−	−	−	−	+	−	−	+	−	−	NT	−
**Cerebellum**													
Gliosis	−	−	−	−	−	+	+	−	+	−	+	NT	−
Perivascular cuffing	−	−	−	+	−	+	+	−	+	−	−	NT	−
Purkinje cell necrosis	−	−	−	+	+	−	+	+	+	−	+	NT	+
Purkinje cell disappearance	+	+	+	+	+	+	+	+	+	−	−	NT	−
Endothelial hypertrophy	+	+	+	+	+	+	+	+	+	+	+	NT	+
**Optic lobe**													
Neuronal necrosis	+	+	+	−	−	−	−	+	+	−	+	NT	NT
Gliosis	−	+	+	+	−	+	+	−	+	−	+	NT	NT
Perivascular cuffing	−	−	−	−	+	−	+	−	+	−	−	NT	NT
Endothelial hypertrophy	+	+	+	+	+	+	+	+	+	+	+	NT	NT
Glial necrosis	−	−	−	+	−	+	−	−	−	−	−	NT	NT
**Spleen**													
Capsular thickening	+	+	+	+	−	+	−	+	−	NT	−	NT	−
Lymphoid depletion	+	+	+	+	+	+	+	+	+	NT	+	NT	+
Haemosiderosis	+	+	+	+	+	+	−	−	−	NT	−	NT	+
Granulocytic infiltration	+	+	+	−	−	−	+	−	+	NT	−	NT	−
Lymphoid necrosis	−	−	+	+	+	−	−	−	−	NT	+	NT	−
Endothelial swelling	−	−	−	−	−	+	−	+	+	NT	−	NT	−
Vascular wall thickening	+	−	−	−	−	−	−	+	+	NT	−	NT	−
**Large intestine**													
Epithelial necrosis	−	−	+	+	−	−	−	−	−	NT	NT	NT	NT
**Liver**													
Inflammatory infiltrate	−	+	+	+	+	+	−	+	+	−	−	−	−
Haemosiderosis	+	+	+	+	+	+	−	−	−	−	−	−	+
Hepatocellular necrosis	−	−	−	+	−	−	−	−	−	−	+	−	−
**Pancreas**													
Acinar necrosis	+	−	−	+	−	NT	−	−	−	NT	NT	NT	−
**Kidney**													
Inflammatory infiltrate	+	+	−	+	+	−	−	−	+	−	+	−	−
Tubular degeneration	−	−	−	+	+	+	+	+	+	−	−	−	−
Tubular necrosis	−	−	+	+	−	−	+	−	+	+	+	−	+
**Heart**													
Inflammatory infiltrate	−	−	+	+	−	+	−	−	+	+	+	NT	+
Miofibrilar degeneration	−	−	+	+	−	+	−	−	+	+	+	NT	−
Miofibrilar necrosis	−	−	+	+	−	−	−	−	−	−	−	NT	−
**Lung**													
Inflammatory infiltrate	−	+	−	+	+	−	−	+	+	−	−	−	−
Septal epithelial cells necrosis	−	−	−	−	+	−	+	−	−	−	−	−	−
**Skin**													
Inflammatory infiltrate	−	+	+	−	+	+	−	−	−	−	−	−	NT

+ Presence of lesion.

– Absence of lesion.

NT Tissue not tested.

**Figure 7:**
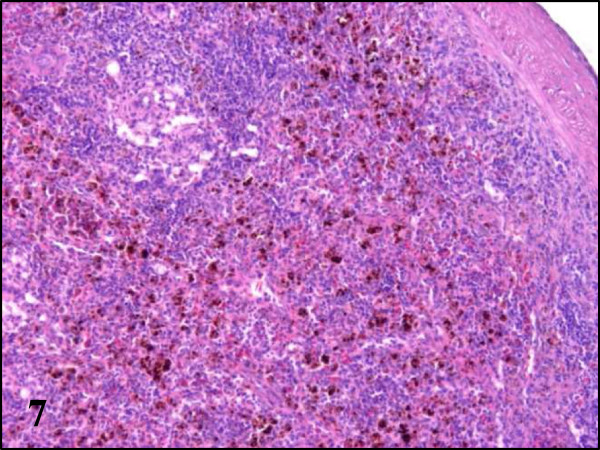
**Haemosiderosis in spleen section of red-legged partridges infected with BAGV.** HE ×100.

**Figure 8:**
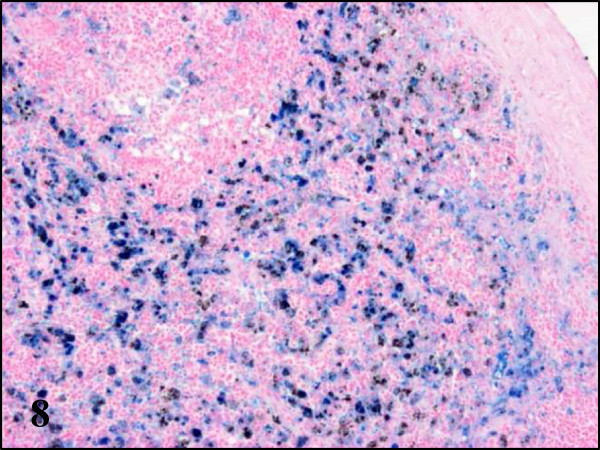
**Haemosiderosis in spleen section of red-legged partridges infected with BAGV.** Perls’ stain, ×100.

**Figure 9:**
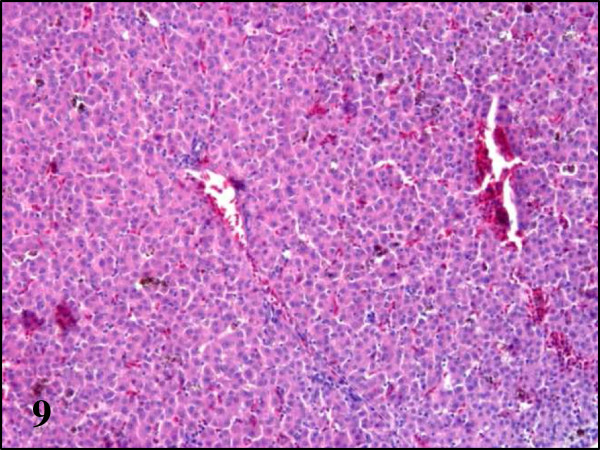
**Haemosiderosis in liver section of red-legged partridges infected with BAGV.** HE, ×100.

**Figure 10:**
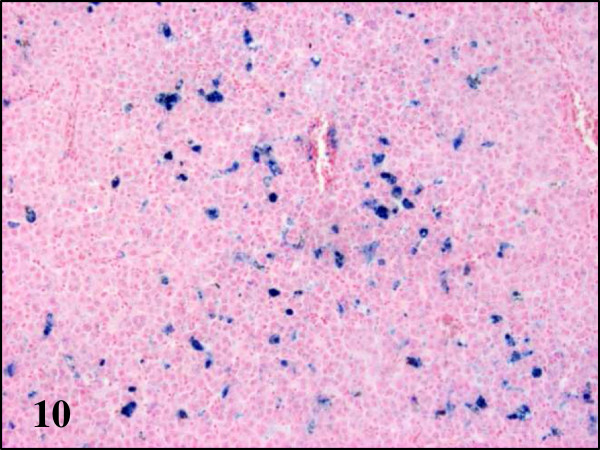
**Haemosiderosis in liver section of red-legged partridges infected with BAGV.** Perls’ stain, ×100

While partridges showed severe haemosiderosis in the spleen and the liver, this was absent in pheasants and less evident in wood pigeons (Table 2, Figures 7 and 8). All examined partridges and to a lesser extent wood pigeons, had Kupffer cells and hepatocytes in the liver and macrophages in the spleen heavily laden with brown pigment. Using Perl’s stain, this brown pigment was shown to contain iron thus indicating that in fact, it corresponds to haemosiderin (Figures 9 and 10). In pheasants no iron/haemosiderin was evidenced in the liver or spleen using this technique.

**Figure 11:**
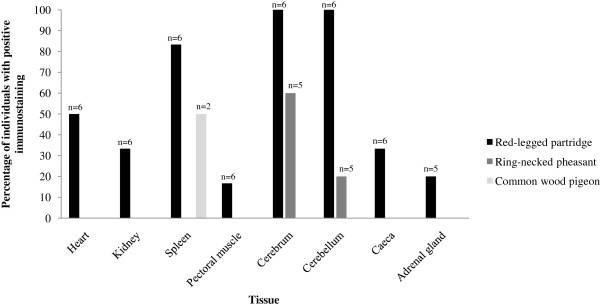
**Distribution of positive flavivirus (presumptive BAGV) immunostaining in naturally BAGV infected red-legged partridges, ring-necked pheasants and common wood pigeons.** Each column represents the percentage of individuals that showed positive immunostaining in each organ and “n” is the number of individuals in which each tissue was tested.

**Figure 12:**
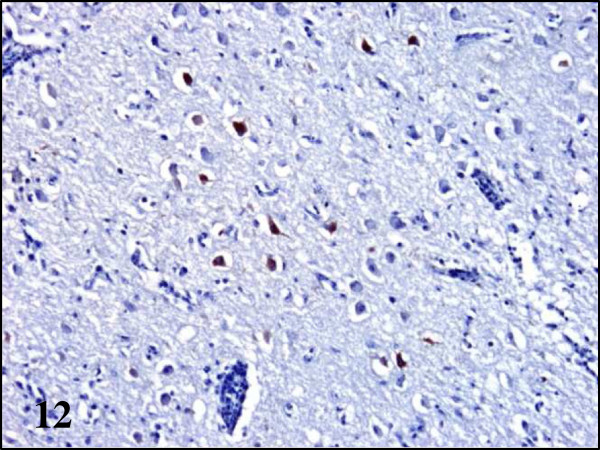
**Viral antigen distribution in the brain of BAGV infected game birds.** Cerebrum, ring-necked pheasant. Labeling of BAGV antigen in the cytoplasm of neurons. Immunohistochemistry for detection of flavivirus antigen, ×100.

**Figure 13:**
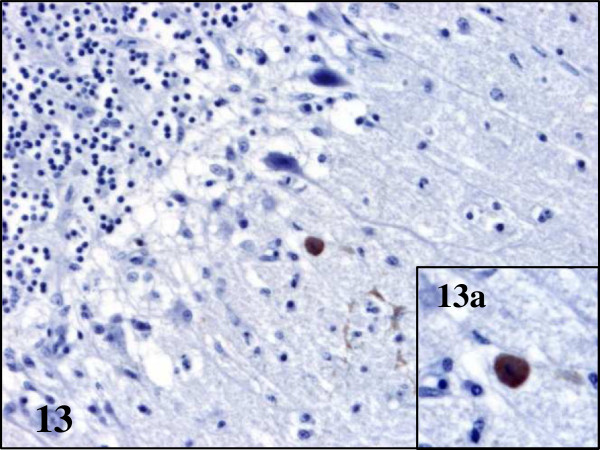
**Viral antigen distribution in the brain of BAGV infected game birds.** Cerebellum, ring-necked pheasant. Labeling of BAGV antigen in the cytoplasm of Purkinje cells (**13a**). Immunohistochemistry for detection of flavivirus antigen, ×200; ×400 (13a).

**Figure 14:**
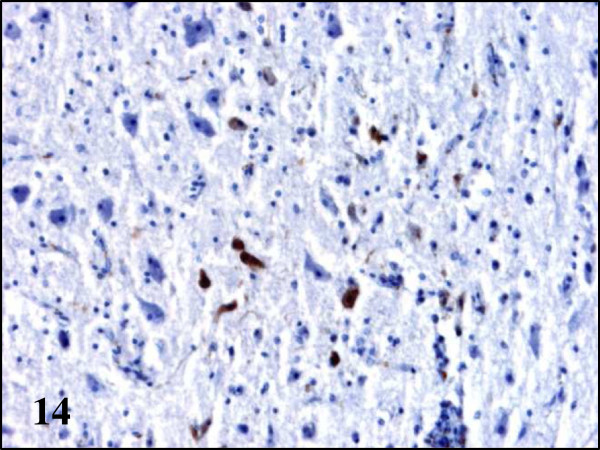
**Viral antigen distribution in the brain of BAGV infected game birds.** Cerebrum, red-legged partridge. Labeling of BAGV antigen in the cytoplasm of neurons and capillary endothelial cells. Immunohistochemistry for detection of flavivirus antigen, ×200.

**Figure 15:**
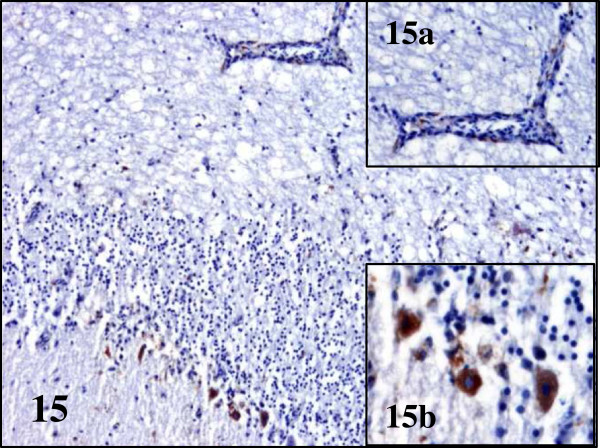
**Viral antigen distribution in the brain of BAGV infected game birds.** Cerebellum, red-legged partridge. Labeling of BAGV antigen in the capillary endothelial cells (**15a**) and cytoplasm of Purkinje cells (**15b**). Immunohistochemistry for detection of flavivirus antigen, ×100; ×400 (15a and b).

Using immunohistochemistry, BAGV antigen was detected in numerous organs of the partridges, especially in capillary endothelial cells, while in the wood pigeon it was only detected in the spleen and in the pheasants in the central nervous system (Figure 11). In pheasants, only the cytoplasm of neurons and glial cells of the thalamus and optic lobe and Purkinje cells of the cerebellum were found to contain BAGV antigen (Figures 12 and 13, 13a). In partridges endothelial cells of capillaries of the cerebrum, cerebellum, spleen, heart, kidney, pectoral muscle, caeca and adrenal gland contained BAGV antigen in their cytoplasm. Other cells that tested positive were neurons and glial cells of the cerebrum, Purkinje cells of the cerebellum, cardiomyocytes of the heart and endothelial cells of the glomerular mesangium in the kidney (Figures 14, 15, 15a and b, 5a and 6a). One of the wood pigeons had BAGV antigen in capillary endothelial cells in the spleen. As swabs and tissues tested by real time RT-PCR were negative for WNV, and the only flavivirus identified was BAGV, we assumed that positive labelling in immunohistochemistry preparations indicated presence of BAGV antigen.

Incidental findings included avian tuberculosis, anthracosis and coccidian oocysts. Two of the pheasants and one of the wood pigeons had lesions compatible with avian tuberculosis, in which presence of acid resistant bacilli was confirmed by Ziehl-Neelsen stain. All of the partridges and one pheasant had brown pigment and crystalline structures within the cytoplasm of peribronchial macrophages in the lungs, compatible with anthracosis. Finally, two of the partridges showed less than five coccidian oocysts/cross sections in the intestinal mucosal epithelium of the large intestine.

## Discussion

The BAGV outbreak in 2010 in Spain is the first documented occasion in which this virus has caused disease and mortality in birds, with a strikingly most severe effect on a game bird species, the red-legged partridge [1]. Recently authors reporting on the parallel outbreak of WNV in horses in the same region made the hot summer and high mosquito abundance responsible for both outbreaks [10]. While several cases of WN fever in horses were notified, no new mortality events among game birds were detected during 2011.

Pathology due to BAGV infection had previously not been described in any species as the presence of neutralizing antibodies against BAGV in persons with acute encephalitis in India, could not link the infection clearly to disease symptoms [11]. However, based on sequence analysis, BAGV has been shown to be synonymous to Israel turkey meningoencephalitis virus (ITV) [12], which causes a disease characterized by nonpurulent meningoencephalitis with lymphocytic perivascular infiltrates and focal myocardial necrosis in turkeys in Israel and South Africa, and is controlled by vaccination with live attenuated vaccines [13-15]. The close genetic relationship between the two viruses may mean that BAGV is similarly pathogenic to at least some bird species [3].

A local hunting estate in the area of the outbreak that conducts direct transect counts on game birds prior to and after the hunting season, reported reduction of red-legged partridge numbers by 86% and in ring-necked pheasants by 29%. Due to the high mortality in red-legged partridges, only ring-necked pheasants were hunted in winter 2010/2011. Hunting bag data from the same hunting estate, showed a reduction in the female/male ratio with respect to previous years (2.4 females/male as opposed to 4 females/male), suggesting that female pheasants were more affected than males.

The degree to which the impact of BAGV in red-legged partridge populations is higher than in ring-necked pheasants and other birds is evidenced by differences in mortality and in the incidence of concomitant disease observed. In all examined partridges BAGV infection appeared to be the primary cause of death (2/6) or disease (4/6), while two of five pheasants and one of the two wood pigeons had severe advanced lesions of concurrent chronic disease (avian tuberculosis). The reason of the higher reduction in numbers in female pheasants, as opposed to males is unclear. With view to a potential higher impact of BAGV, disease due to ITV has also been reported to be more severe and frequent in female than in male turkeys [15]. However other effects such as higher susceptibility of female pheasants to predation, other diseases, or toxic substances employed in agriculture in the area cannot be ruled out.

Differences in the pathogenicity of BAGV for the three species could partly be explained by the differences observed in the distribution and severity of lesions, distribution of viral antigen, and the severe haemosiderosis in partridges, that was moderate in wood pigeons and absent in pheasants. In partridges, BAGV apparently has a wide tropism, targeting different cell types, but especially capillary endothelial cells. In pheasants, neurotropism appears to be somewhat more important while in pigeons only endothelial cells of splenic capillaries seemed to contain BAGV antigen.

One of the most well studied flaviviruses that is known to cause disease in birds is WNV, of which information is available on pathology in naturally infected humans, horses and birds as well as experimental avian and mouse models [9,16-18]. WNV is known to vary greatly in its virulence in avian species although the mechanism and reasons are still poorly understood [9,19,20]. As an example, both endothelial and neural tropism has been described in native North American avian species after natural WNV infection [21-23]. Also in humans and mouse models WNV has been shown to have a diverse cell tropism, leading to a variety of lesions and clinical manifestations [18,24].

The severe haemosiderosis in red-legged partridges that is the most striking difference to lesions due to BAGV in ring-necked pheasants has previously been described in birds naturally infected by WNV. Hepatic haemosiderosis is most well known as associated to iron overload in captive wild forest birds but is also frequently associated to haemolytic processes in infectious disease [25]. Haemosiderosis and haemorrhage in relation to WNV has been described previously in the spleen and liver of naturally infected North American passeriformes such as the blue jay (*Cyanocitta cristata*) and the house sparrow (*Passer domesticus*) as well as raptors and owls from the North American continent [26-28]. It has also been described in wild turkeys (*Meleagris gallopavo*) [29], but in an outbreak in farmed chukar partridges (*Alectoris chukar*) and Impeyan pheasants (*Lophophorus impeyanu*s), only erythrocytophagocytosis was reported in the spleen, while haemosiderosis was not observed [30]. Also, experimental infection of red-legged partridges with Mediterranean WNV isolates did not lead to a noticeable degree of haemosiderosis (the authors, data not published). With view to WNV-associated haemolysis in other species, a few cases of WNV-associated haemorrhagic fever in humans have been described [31]. A recent study has associated sequence signatures in the envelope protein of human pathogenic flaviviruses with the primary syndrome that they produce (encephalitis or haemorrhagic disease) [32]. These authors speculated that the electrostatic charge differences caused by the presence of either glycosilated asparagine (Asn, haemorrhagic viruses) or Aspartic acid (Asp, encephalitic viruses) at position 67 of the domain II of the envelope protein could be responsible for the phenotype and related disease syndrome. It would be of interest to further characterize the outbreak-related virus, in order to study these and other features.

In conclusion, BAGV is more pathogenic for red-legged partridges than for ring-necked pheasants and common wood pigeons, and causes a severe haemolytic process in this species. Further (experimental) studies will be necessary to determine the factors that trigger BAGV susceptibility and pathogenesis of the infection in red-legged partridges.

## Competing interests

The authors declare that they have no competing interests.

## Authors’ contributions

VG, JAO, AVGG and MDM collected and processed the samples. JAO collected and interpreted data from direct counts. AVVG, MDM and IGFM did the PCRs, carried out BLAST searches, interpreted the sequence data and wrote the sections of the paper devoted to clinical signs and molecular detection of viral antigen. VG and UH examined and interpreted the histologic sections and VG wrote the draft manuscript. CG participated in the design of the study and helped with early drafts of the paper. JF helped with molecular sequence analysis. UH conceived of the study, participated in its coordination and helped to draft the manuscript. All authors read and approved the final manuscript.
